# Anthropogenic Noise Source and Intensity Effects on Mood and Relaxation in Simulated Park Environments

**DOI:** 10.3389/fpsyg.2020.570694

**Published:** 2020-10-14

**Authors:** Jacob A. Benfield, Gretchen A. Nurse Rainbolt, Lucy J. Troup, Paul A. Bell

**Affiliations:** ^1^Department of Psychological and Social Science, Pennsylvania State University, Abington, PA, United States; ^2^Department of Psychology, Colorado State University, Fort Collins, CO, United States; ^3^School of Education and Social Sciences, University of the West of Scotland, Paisley, SC, United Kingdom

**Keywords:** soundscapes, aircraft, transportation noise, national parks, stressors

## Abstract

Research on human caused sound has shown a wide range of effects in outdoor environments as well as laboratory simulations of those environments. Aircraft noise, ground traffic, and human voices have all been shown to lower scenic evaluation ratings and influence individual reports of affective state. However, previous research has relied entirely on pre-post measures of affect and psychological state rather than more momentary assessments. The current project utilized a time series of 15 measurements of overall mood and relaxation collected during a 30-min period during which participants (*N* = 229) were exposed to randomized volume levels of natural sounds, natural sounds with aircraft noise, natural sounds with ground traffic, or natural sounds with human voices added. Results supported previous findings with significant sound type X volume interactions showing differing rates of decline for both outcomes. Natural sounds did not relate to the diminishing effects observed for the three anthropogenic sound conditions.

## Introduction

Noise, defined as *unwanted* or *harmful* sound, is often considered an ambient stressor by environmental psychologists because noise can place demands on us to cope or adapt while simultaneously influencing our psychological well-being (Seidman and Standring, 2010). For example, Evans et al. (2001) showed that children chronically exposed to noise sources such as road traffic at average sound levels greater than 60 dB had elevated systolic blood pressure and higher overnight urinary cortisol levels when compared to children with day-to-day exposure at lower sound intensity levels (<50 dB). The high-exposure children also self-reported higher perceived stress levels and demonstrated high physiological reactivity in laboratory manipulations, suggesting that noise exposure was interfering with relaxation while promoting stress and heightened physiological arousal. In addition to physiological stress responses, noise also impacts perceptual and psychological reactions to the environment. For example, research has found that positive affective states are compromised when specific sounds are perceived as “noise” (e.g.,Tarrant et al., 1995).

### Noise and Natural Environments

The United States National Park Service (NPS) has jurisdiction over hundreds of natural, historical, and cultural sites throughout the United States of America and has been charged with both preserving the natural ecosystem for future generations and allowing for public visitation and recreation (Yellowstone Act of 1872, 1872; National Wilderness Preservation Act of 1964, 1964). Additional mandates requiring the federal government to assess and monitor soundscapes in National Parks demonstrate recognition of the ambient acoustic environment as one of the potential elements influencing both wildlife and visitor experiences (Noise Control Act of 1972, 1972). Moreover, other legislation (e.g., National Parks Air Tour Management Act of 2000, 2000) has been enacted to specifically target the management and study of specific noise sources such as aircraft overflights or recreational vehicles such as snowmobiles.

To facilitate these research and management goals, scientists have identified key indicators used to evaluate various aspects of soundscapes in recreational settings. Some well-established factors used to evaluate visitor experience have been based on outcomes related to stated preferences (Driver et al., 1987), landscape assessment (Daniel 2001), and affective responses to natural environments (Kaplan, 1995). For example, in the context of landscape assessment, Mace et al., 1999 found that the evaluations of scenic park landscapes along 8 dimensions were significantly lower in the presence of both 40 and 80 db helicopter overflight sounds. Follow-up research showed the effect to be salient across different noise source attributions (Mace et al., 2003) and additional noise source types such as human voices and automobile traffic (Benfield et al., 2010).

### Noise and Affective Responses to Natural Environments

At the same time, the introduction of noise to a landscape not only impacts scene assessments, but also impacts the affective state of those engaged in the assessing. However, the results of those tests have been less consistent and harder to interpret collectively. For instance, Mace et al. (1999, 2003) and Benfield et al. (2010) each found that the addition of noise diminished landscape evaluations, but all also showed discrepant findings when it came to measures of affect. In those series of related studies, each found different combinations of changes in positive and negative affect, in spite of all three using comparable methodologies and stimuli.

Specifically, all three studies utilized the Positive and Negative Affect Schedule (PANAS; Watson and Clark, 1999) and a pre-post procedure for measuring change from exposure. All three studies required participants to evaluate various landscapes on a large screen, projected in a dark room with sounds presented at comparable intensity levels using surround sound speakers. Each study session lasted approximately one hour, with half of that time spent on the noise exposure and scenic evaluation task. Finally, all three studies demonstrated a decrease in positive affect in the presence of noise, although Benfield et al. (2010) showed the decrease in positive affect for all conditions, including the natural and control sound conditions, suggesting something other than the experimental manipulation.

In addition to some variance in positive affect findings, the results for negative affect were also inconsistent across conditions and studies. The original Mace et al. (1999) study showed no effect of noise on negative affect and attributed this finding, when paired with the loss of positive affect, as indicating a decrease in pleasure but not an increase in annoyance. However, the follow-up work in 2003 by Mace and colleagues did show increased negative affect, but not for all noise conditions. It was suggested that this effect may be an artifact of methodology or the result of different situational attributions assigned to the noise. Benfield et al. (2010) also failed to show an effect on negative affect but because of the global decrease in positive affect, suspected another factor at play. Specifically, Benfield et al. (2010) argued that the methodology, which was identical to Mace et al. (1999) and very similar to Mace et al. (2003), was causing participant fatigue and boredom which would explain a lowering of positive affect as well as the null effect on negative affect. Such a possibility has not been directly tested in laboratory soundscape research, yet could alter the interpretation of previous work and better inform future simulation work on this key management indicator.

### The Current Study

Though previous research showed a relationship between noise exposure and mood and stress, the temporal evolution of these responses has not been documented. Substantial intervals between affective assays introduced potential confounding explanations, and may explain some of the disparate results. Those previous effects are often shown cross-sectionally or with simple pre-post measures of mood separated by up to an hour of time. The current study aimed to rectify that confound in methodology by measuring affective valence and arousal levels at regular intervals, throughout the scenic evaluation task, rather than in a pre-post framework. Additionally, the current study included direct self-report measures of both fatigue and effort, also recorded at regular intervals throughout the task, to control for additional factors that could explain prior findings in this domain.

Moreover, laboratory based research in this area is not always in agreement concerning the size, cause, or direction of effect (e.g., Mace et al., 1999 compared to Benfield et al., 2010) and often fails to test the effect of sound intensity alongside sound type. Specifically, even though participants in previous studies were exposed to different sound intensities during the evaluation task, the pre-post measurement procedure prevented testing the effect of intensity on mood. As such, those previous projects demonstrated clear connections between sound intensity level and scenic evaluations, but never to changes in affective state. Therefore, it was also the purpose of the current study to examine the interaction of sound intensity and sound type on affective valence and arousal. That is, the purpose of the current project was to test the robustness of the previous findings on natural soundscapes’ effect on mood and stress with a previously unutilized methodology and set of measurements.

## Materials and Methods

### Overall Design

A 4 (sound type) x 3 (sound intensity) mixed factorial timeseries design was utilized in this study. One of four soundscape conditions (between-subjects factor) were randomly administered to participants who each experienced three randomly presented sound intensity levels for that soundscape (within-subjects factor). Assessments of mood, relaxation, fatigue, and effort were made every two minutes throughout the 30-min landscape evaluation task for a total of 15 individual measurements, five for each of the sound intensity levels.

### Participants

A total of 229 undergraduates (140 females; 89 males) participated in the research as partial fulfillment of a compulsory course research requirement. Participants were about 19 years old (*M* = 19.30, *SD* = 2.20, Range = 18 – 43) and reported visiting an average of 4 or 5 United States national parks in their lifetimes (*M* = 4.78, *SD* = 3.56, Range = 0 – 19). The majority of participants were of European descent (87%).

### Materials and Measures

#### Predictor and Outcome Measures

The Weinstein Noise Sensitivity Scale (WNS) measures individual sensitivity to unwanted sounds or noises (Weinstein, 1978). It consists of 21 items rated on a 6-point scale ranging from ‘strongly disagree’ to ‘strongly agree.’ Summation of items creates a single score for overall noise sensitivity (α = 0.84). Previous research has shown noise sensitivity to be an important covariate when assessing the effects of noise on humans (Ellermeier et al., 2001; Miedema and Vos, 2003) and to be important in measuring recreation noise acceptability (Benfield et al., 2014).

Visual analog scales (VAS) were used for the measurement of both outcome variables (overall mood and overall level of relaxation) as well as two control variables (participant level of fatigue and overall effort). These VAS measures consisted of a five-inch (12.7 cm) horizontal line representing the continuum from “very low” to “very high” (or “very negative” to “very positive” for the mood rating). Participants responded by making a vertical mark along the continuum, and scores were calculated by measuring the distance of the vertical mark from the left edge of the continuum in 1/8^*th*^ inch increments (0.3 cm). This created a range of 0–40 points which provided a continuous, ratio distribution of the response data while helping to reduce range restriction sometimes observed when responding with discrete values (e.g., the typical 1–7 range used in Likert-type response measures). Research on VAS rating scales shows them to be a sensitive, valid, and reliable technique for obtaining participant responses across a range of phenomena including pain, attractiveness, self-esteem, and mood, especially when the time between responses is limited (Folstein and Luria, 1973; Price et al., 1983; Brumfitt and Sheeran, 1999; Grant et al., 1999; Rankin and Borah, 2003; Hasson and Arnetz, 2005).

#### Soundtracks

Using an acoustics database maintained by the NPS, four different sound recordings were used for the auditory manipulation. The natural sound condition (wind through foliage with mixed bird calls) was used as a baseline for the other three sound conditions: natural with voices, natural with ground traffic, and natural with air traffic. Each sound clip was created by adding isolated recordings of the noise (e.g., voices) to the natural baseline. For these sound conditions, the added element was present on an almost continuous basis with the longest gap between noise events being less than 10 *s.*

#### Visual Stimuli

A set of 30 scenes was assembled as the visual stimuli presented for rating. The first 5 scenes were practice slides to familiarize the participants with the procedure, rating sheet, and slide timings. The remaining 25 slides were target slides representing five scenes each from five national parks—Yellowstone, Olympic, Saguaro, Grand Canyon, and Everglades—which were chosen from a set of high-resolution photographs taken within each park. While the order of each park was presented randomly, the five scenes within each park were shown consecutively in a non-randomized order.

### Procedure

Participants signed up for the study using an online recruitment website associated with an introductory psychology course and attended a single, one-hour research session. After completing an initial informed consent sheet, participants were then randomly assigned to one of the four sound conditions. All experimental sessions were conducted with participants seated 10 ft (3 m) away from a 6 × 6 ft (1.8 m × 1.8 m) screen mounted at the front of an 18 × 18 ft (5.5 m × 5.5 m) room. Scenes were presented on the screen via computer projector, and sounds were presented using a 4-channel surround sound system placed in the corners of the room.

A brief demographic questionnaire containing the WNS and other measures was presented at the beginning of the larger research packet. Upon completion, participants were then given instructions concerning the scenic evaluation task. The scenic evaluation task required participants to rate five practice slides and then view the set of 25 target slides three separate times in each session (20 *s* per slide; 80 slide ratings total). Each of the three runs of the 25 target slides was accompanied by one of the three sound levels: (1) control, or no added sounds to the 40–45 dB(A) background from the room; (2) low volume added sounds of 40–45 dB(A); or (3) high volume added sounds of 60–65 dB(A). The three sound levels were presented in one of four random orders with gradual changes in sound intensity occurring over a 20 *s* period at the change of conditions.

The VAS ratings for mood, relaxation, fatigue, and effort were presented after every set of five slides starting after the five practice slides. Because of the number of slide sets and the timing of the sets, a total of 15 VAS measurements were taken over the course of the 30-min scenic evaluation task with measurements spaced 2 min apart. At the conclusion of the evaluation task, participants were fully debriefed with regard to the purposes and methods of the project and given course research credit for compensation.

### Data Analysis Strategy

Growth modeling is a multilevel data analysis technique that allows researchers to examine longitudinal or time series data for both intraindividual change (how do people change over time) and interindividual differences in change (how do people differ in how they change over time; Henry and Slater, 2007). In a multilevel framework, level 1 consists of the multiple measurement occasions recorded for an individual; level 2 is made up of the individuals themselves. Within the current study, growth modeling allows for the examination of change within individuals based on the length of time they have been participating, the changes in volume level that have occurred, or their individual level of fatigue/effort (level 1). It also allows for the simultaneous assessment of differences in change between sound exposure conditions or noise sensitivity scores (level 2).

For both outcomes—overall mood and relaxation—a series of six growth models were created. The first three (Models A – C in Tables 1, 2) represent unconditional models designed to provide baseline measures of variance and model fit as well as to assess the best way to model the effect of time (i.e., measurement occasion) onto the data. The remaining three models (D – F in Tables 1, 2) represent conditional models in which the effects of the experimental manipulations (Model D), individual noise sensitivity (Model E), and participant fatigue/effort (Model F) on the outcome variable are modeled. For the purposes of brevity, only conditional models are discussed extensively. It suffices to say testing of unconditional models A-C showed that significant amounts of both within- and between-person variance existed (ICC = 0.601 and 0.608 for mood and relaxation, respectively), and that quadratic models were preferable to linear models of change over time.

**TABLE 1:** Model summaries for VAS mood outcome scores.

	Model A (Means)	Model B (Linear)	Model C (Quadratic)	Model D (Experimental)	Model E (WNS)	Model F (Fatigue/Effort)
**Fixed Effects (Mood)**						
Initial Status						
Intercept	27.966**	28.974**	30.111**	29.778**	29.748**	20.708**
Automobile				2.088	2.200	1.590
Auto * Noise sensitivity					–0.124	–0.086
Aircraft				2.786**	2.802**	2.375**
Aircraft * Noise Sensitivity					–0.106	–0.130
Voices				1.167	1.101	1.051
Voices * Noise Sensitivity					−0.224*	−0.197*
Volume Order (C-65-45)				–0.464	–0.298	–0.194
Volume Order (45-65-C)				–0.368	–0.399	–0.280
Volume Order (65-45-C)				–0.201	–0.446	–0.547
Volume(45 dBA)				0.520	0.553	0.618
Volume(65 dBA)				−1.113*	−1.104*	−1.103*
Vol(45 dBA) * Auto				−2.574**	−2.571**	−2.325**
* Aircraft				−3.357**	−3.392**	−3.312**
* Voices				−3.225**	−3.327**	−3.365**
* WNS					−0.055*	−0.048*
Vol(65 dBA) * Auto				−3.306**	−3.307**	−2.792**
* Aircraft				−4.639**	−4.645**	−4.247**
* Voices				−4.400**	−4.445**	−4.386**
* WNS					–0.049	–0.053
**Fixed Effects (Cont.)**						
Noise Sensitivity					0.016	0.011
Fatigue (Level 1)						−0.093**
Effort (Level 1)						0.287**
Fatigue (Level 2)						−0.175**
Effort (Level 2)						0.372**
Rate of Change						
Time		−0.145**	−0.669**	−0.254*	−0.248*	−0.279**
Quadratic Time						
Time^2^			0.037**	0.010	0.010	0.014*
**Variance Components**						
Level 1 – Within	29.883**	29.622**	26.107**	25.994**	25.986**	23.782**
Variance Explained	2.45%	6.62%	1.82%	6.30%	19.82%	
Level 2 – Between	45.070**	43.496**	42.171**	41.042**	36.830**	26.581**
Variance Explained	3.49%	3.07%	2.67%	10.24%	27.77%	
**Model Fit**						
Log Likelihood (CF for MLR)	−11940.076(4.762)	−11473.485(2.678)	−11343.224(2.154)	−11100.654(1.532)	−11086.625(1.468)	−10933.910(1.537)
Parameters	3	6	10	33	39	43
Chi-square Change (TRD)		1571.013	190.4401	384.554	25.14158	138.2183
*p*-value		<0.001	<0.001	<0.001	<0.001	<0.001

*p < 0.05; **p < 0.01. Referent Groups: Natural Sounds; Control-45–65 dBA Volume Order; 0 dB Volume (control).

**TABLE 2:** Model summaries for VAS relaxation outcome scores.

	Model A (Means)	Model B (Linear)	Model C (Quadratic)	Model D (Experimental)	Model E (WNS)	Model F (Fatigue/Effort)
**Fixed Effects (Relax)**						
Initial Status						
Intercept	24.841**	25.427**	26.879**	27.061**	26.988**	17.597**
Automobile				1.086	1.185	0.585
Auto * NoiseSensitivity					–0.111	–0.072
Aircraft				1.380	1.405	0.920
Aircraft * NoiseSensitivity					–0.106	–0.118
Voices				1.179	1.044	0.845
Voices * NoiseSensitivity					−0.259**	−0.240*
Volume Order (C-65-45)				0.409	0.692	0.939
Volume Order (45-65-C)				1.044	1.017	1.110
Volume Order (65-45-C)				0.680	0.607	0.583
Volume(45 dBA)				–0.388	–0.387	–0.329
Volume(65 dBA)				−2.555**	−2.553**	−2.558**
Vol(45 dBA) * Auto				−2.118**	−2.092**	−1.951**
* Aircraft				−4.337**	−4.327**	−4.310**
* Voices				−4.108**	−4.132**	−4.167**
* WNS					–0.040	–0.034
Vol(65 dBA) * Auto				−3.118**	−3.118**	−2.763**
* Aircraft				−6.088**	−6.082**	−5.787**
* Voices				−4.436**	−4.453**	−4.364**
* WNS					–0.036	–0.040
**Fixed Effects (Relax)**						
WNS					0.005	–0.001
Fatigue (Level 1)						−0.101**
Effort (Level 1)						0.232**
Fatigue (Level 2)						−0.177**
Fatigue (Level 2)						0.384**
Rate of Change						
Intercept		–0.086	−0.755**	–0.147	–0.143	–0.160
Quadratic Time						
Time^2^			0.048**	0.006	0.006	0.009
**Variance Components**						
Level 1 – Within	37.546**	37.431**	33.947**	33.677**	33.675	31.706**
Variance Explained	1.67%	4.34%	1.43%	5.10%	14.31%	
Level 2 – Intercept	58.282**	56.798**	56.191**	55.171**	50.632**	40.536**
Variance Explained	2.54%	1.09%	1.81%	8.19%	19.90%	
**Model Fit**						
Loglikelihood (CF for MLR)	−12366.300(3.317)	−11938.664(2.328)	−11790.389(1.759)	−11501.677(1.317)	−11490.211(1.287)	−11393.431(1.373)
Parameters	3	6	10	33	39	43
Chi-square Change (TRD)		638.7394	327.4986	513.3451	20.4385	87.5243
*p*-value		<0.001	<0.001	<0.001	0.002	<0.001

*p < 0.05; **p < 0.01. Referent Groups: Natural Sounds; Control-45–65 dBA Volume Order; 0 dB Volume (control).

All analyses were conducted using the SAS PROC MIXED procedure (Singer, 1998; Singer and Willett, 2003). Tests for multicollinearity showed variance inflation factor (VIF) values within an acceptable range (VIF = 1.06–2.49). Variance explained values are based on pseudo-R^2^ statistics for both levels of the model (Singer and Willett, 2003).

## Results

### Overall Mood

For all three conditional models, the inclusion of the added parameters significantly improved model fit and supported previous research on both sound type and sound volume. The inclusion of the experimental variables (i.e., sound type, sound volume, sound order, and a sound type X sound volume interaction term) showed no main effect for sound order, sound type, or the low-volume condition. However, the model did demonstrate a negative effect for the high-volume condition, and all three noise types significantly interacted with both the high- and low-volume conditions (Model D in Table 1). Noise significantly decreased mood ratings, but the size of the detriment varied depending on the type of sound and the volume level of the sound. High-volume exposure was always more detrimental than low-volume exposure with human voices (β = −3.23 for low volume; −4.40 for high volume) and aircraft noises (β = −3.36 for low volume; −.64 for high volume) having a larger effect than automobile traffic noise (β = −2.57 for low volume; -3.31 for high volume). The inclusion of the experimental variables explained an additional 1.82% of variance in scores across time points and 2.67% of the variance in individual average mood scores.

**FIGURE 1:**
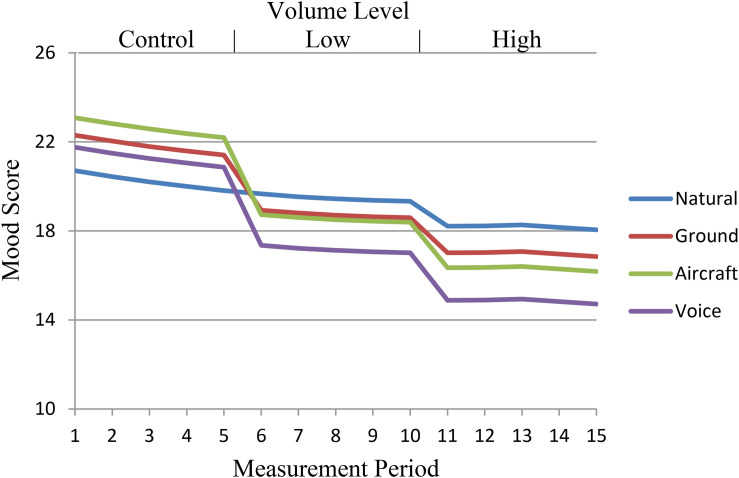
Prototypical change in mood score based on sound type, volume level, and measurement occasion assuming average levels of noise sensitivity, fatigue, and effort.

The pattern of results shown in the first conditional model persisted through the other two conditional models. Significant sound type X volume level interactions showed high volume levels to be more problematic than low volume levels with human voices and aircraft noise being more bothersome than automobile noise. The addition of the individual noise sensitivity covariate explained 6.30% of variance across measurement occasions and 10.24% of average mood score variance (Model E in Table 1). While no main effect for noise sensitivity was shown, a significant interaction with the low volume condition (β = 0.06) showed that higher sensitivity to noise related to a larger negative effect in the low volume condition. That interaction was not significant for the high-volume condition. A similar interaction existed between noise sensitivity and the human voices condition. Greater sensitivity to noise related to lower mood scores when exposed to human voices (β = −0.22); the effect was not shown for aircraft or automobile noises. The fatigue and effort covariates also explained large portions of remaining variance both across measurements and in overall scores (19.82% and 27.77%, respectively), with both covariates having significant main effects on mood (Model F in Table 1). Higher than average fatigue related to decreased mood scores (β = −0.09 within; -0.18 between) while above average effort related to improved mood scores (β = 0.28 within; 0.37 between). All totaled, the addition of the experimental parameters, noise sensitivity, fatigue, and effort into the model (Model F) explained 26.24% of the variance observed across measurement occasions, and 36.90% of the variance in average mood scores across individuals compared to the unconditional quadratic model (i.e., compared to changes occurring only due to time; Model C). Figure 1 displays prototypical mood trajectories based on the average score of all covariates—WNS, fatigue, and effort—across time, volume levels, and sound conditions based on the parameters given in the full model (Table 1, Model F).

### Level of Relaxation

Similar to the findings related to overall mood scores, level of relaxation was significantly affected by the experimental conditions (see Model D in Table 2). Once again, no main effect for sound order, sound type, or low volume was shown but significant sound type X volume level interactions mirrored the pattern of results for mood ratings. Human voices (β = −4.11 for low volume; −4.44 for high volume) and aircraft noise (β = −4.34 for low volume; −6.09 for high volume) were more detrimental than automobile traffic noise (β = 2.12 for low volume; 3.12 for high volume); high volume conditions were more problematic than low volume conditions. The experimental variables explain 1.43% of variability across measurement occasions and 1.81% of variability in average overall scores while significantly improving model fit, *X*^2^ (23) = 513.35, *p* < 0.001.

**FIGURE 2:**
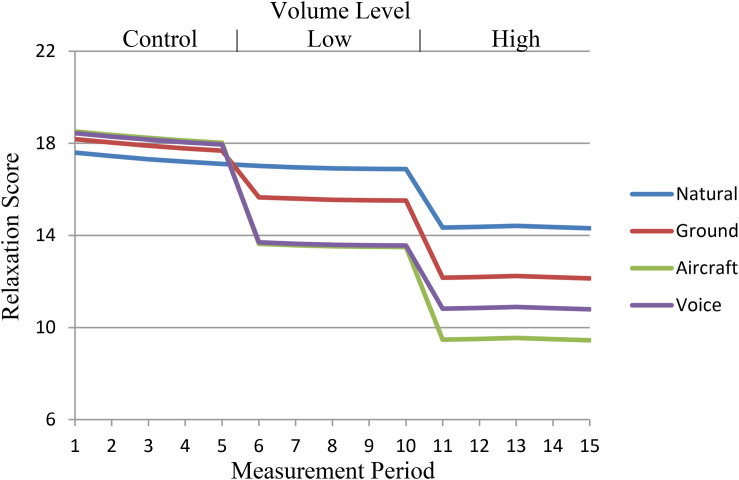
Prototypical change in relaxation score based on sound type, volume level, and measurement occasion assuming average levels of noise sensitivity, fatigue, and effort.

Subsequent models that included the noise sensitivity covariate (Model E in Table 2) and the fatigue and effort covariates (Model F in Table 2) also significantly improved model fit and explained additional within person variance (5.10% for Model E; 14.31% for Model F) and between person variance (8.19% for Model E; 19.90% for Model F). The effects of the experimental variables shown in Model D persisted in the fuller models showing that volume and sound type combine to lower relaxation scores even after controlling for the effects of noise sensitivity and participant fatigue and fluctuation in effort. Figure 2 displays prototypical trajectories for the full model (Model F in Table 2) assuming average levels of noise sensitivity, fatigue, and effort; the full model explained 19.85% of the variability in relaxation across time and 27.81% of the variability in average individual relaxation scores in comparison to the unconditional quadratic model (Model C in Table 2).

## Discussion

Anthropogenic noise, especially at high intensity, decreases individual mood and relaxation while natural sounds have a lessened or null effect depending entirely on sound intensity levels. That noise type by intensity detriment persists even after controlling for individual change across time as well as noise sensitivity, experimental fatigue, and task effort. This finding is consistent with previous research (e.g., Mace et al., 1999; 2003) and builds upon that evidence by showing that the negative effect upon mood or relaxation is not solely an artifact of study design or participant fatigue as others have suggested (e.g., Benfield et al., 2010). It is noteworthy, however, that fatigue and effort were able to account for much more variability in overall mood and relaxation scores than either noise sensitivity or the actual acoustic stimuli alone, suggesting that Benfield and colleagues’ concern about laboratory fatigue or overall effort driving mood findings was not unreasonable. Rarely are such variables directly measured and controlled for in laboratory simulations on noise, and the current data suggest that each can make a substantial impact on findings, especially when measured regularly and alongside certain outcomes. Future research should explore the varied impact of such confounds on similar environmental research and also regularly include controls for them in most, if not all, laboratory simulations and other studies in which exposure is prolonged and participant motivation is potentially less than optimal.

Additionally, noise sensitivity has a measurable influence at low-volume sound levels. High-sensitivity individuals were more disturbed than low-sensitive individuals at low volume levels, but sensitivity had minimal impact at high-volume levels. This information has implications for policy and research. For instance, researchers can better anticipate contexts in which noise sensitivity measurement is more or less crucial to accounting for differences. High intensity sound exposure may not show noise sensitivity effects and therefore may not require controlling for such variables. Likewise, low intensity sounds may only elicit effects when interacting with noise sensitivity and such studies should include measures for sensitivity. In the context of management policy, protected areas visited regularly by persons with higher noise sensitivity (e.g., locations known for unique or subtle sound qualities) may consider more stringent noise abatement strategies, even for sounds that may be physically less intense but reported as problematic. In other words, effective management strategies may require prioritizing subjective visitor ratings of acceptability over objective acoustic measurements of intensity. Similarly, individuals with higher noise sensitivity may be made more aware of how that trait interacts with their perception of sounds as a way to reframe experiences and potentially mitigate conflict with others.

The testing of complex interactions or nuanced effects, such as the role of fatigue on mood across time based on differing sound intensity, is best accomplished under controlled laboratory conditions. The ability to generate causal conclusions regarding naturally occurring phenomenon provides soundscape researchers with a strong foundation on which to build future projects. However, soundscape research and the problems surrounding noise exposure are often more applied and practical in nature. As such, the current project tells us a lot about the role of noise on affective state, arousal, and fatigue in highly controlled situations, but more ecologically valid and intensive field-based studies will be necessary to fully understand the practical implications of these effects.

Ultimately, the repeated measures design of this study provided stronger controls over temporal effects and improved capacity to address differences among subjects. All the effects on mood were shown to occur within very short timeframes (i.e., 2-min evaluation intervals) and can be reversed by lowering sound intensity or removing the stimulus. This has implications for future research and management policy alike. Likewise, these findings demonstrate that growth modeling can detect effects of noise that may be difficult to demonstrate with more traditional statistical techniques. Social science noise researchers may find that more intensive, time-interval based techniques provide stronger evidence of noise impact and can lend itself to more ecologically valid, non-laboratory assessment of noise impacts such as ecological momentary assessments (EMA; Steffens et al., 2017). Such approaches are regularly used by health researchers but less often by environmental psychologists or others within the social science-oriented noise research community. As the current data demonstrate, this relative lack of time-series or momentary assessment is influencing our understanding of the nuance found within human perceptions of soundscape and noise research.

## Data Availability Statement

The raw data supporting the conclusions of this article will be made available by the authors, without undue reservation.

## Ethics Statement

The studies involving human participants were reviewed and approved by Colorado State University Institutional Review Board. The patients/participants provided their written informed consent to participate in this study.

## Author Contributions

All authors listed have made a substantial, direct and intellectual contribution to the work, and approved it for publication. All authors contributed to the article and approved the submitted version.

## Conflict of Interest

The authors declare that the research was conducted in the absence of any commercial or financial relationships that could be construed as a potential conflict of interest.
